# TE studies in Japan: the fourth Japanese meeting on host–transposon interactions

**DOI:** 10.1186/s13100-019-0154-7

**Published:** 2019-03-15

**Authors:** Kenji Ichiyanagi, Kuniaki Saito

**Affiliations:** 1 Laboratory of Genome and Epigenome Dynamics, Department of Animal Sciences, Graduate School of Bioagricultural Sciences, Nagoya University, Furo-cho, Chikusa-ku, Nagoya, 464-8601 Japan; 2 Invertebrate Genetics Laboratory, National Institute of Genetics, Yata 1111, Mishima, Shizuoka, 411-8540 Japan

**Keywords:** Transposable elements, Genome evolution, Chromatin modification, Small RNAs

## Abstract

The fourth Japanese meeting entitled “Biological Function and Evolution through Interactions between Hosts and Transposable Elements (TEs)” was held on August 20–21, 2018 at the National Institute of Genetics (NIG), Mishima, Japan. The meeting was supported by NIG, and its objective was to bring together researchers who study the diverse roles of TEs in genome evolution, as well as host defense systems against TE mobility, such as chromatin modifications, small RNAs, and others. Here, we present the highlights of the talks given by 14 invited speakers.

Organizers: Kenji Ichiyanagi (chief), Kuniaki Saito, and Tetsuji Kakutani.

## Introduction

Transposable elements (TEs) are major components of genomes that stay and/or move around the genome. They play key roles in biological functions and evolution. The interactions between TEs and their hosts have been discussed in a series of meetings entitled “Biological Function and Evolution through Interactions between Hosts and Transposable Elements” that were held at the National Institute of Genetics (NIG), Mishima, Japan. These meetings have provided opportunities for TE researchers working in Japan to gather and exchange their ideas. At the fourth meeting, held on August 20–21, 2018, we had talks given by 14 invited speakers over five sessions that were chaired by **Toshifumi Ishino** (Tokyo Medical and Dental University, Japan), **Yoichi Shinkai** (RIKEN, Japan), **Tomoichiro Miyoshi** (Kyoto University, Japan), **Tetsuji Kakutani** (The University of Tokyo, Japan), and **Kenji Ichiyanagi** (Nagoya University, Japan). The meeting had approximately 50 attendees, the majority of whom contributed to the constructive discussions throughout.

## Highlights of the talks

### TEs in gene regulation

More than half a century ago, Dr. Barbara McClintock noted that gene elements and controlling elements represent two different classes of primary components of the chromosome and that a close relationship exists between them, and today it is widely accepted that TEs have played significant roles in host genome evolution. **Hidenori Nishihara** (Tokyo Institute of Technology, Japan) reported examples of TE exaptation for regulating mammalian gene expression. First, he showed that a cluster of three mammalian TEs, AmnSINE1, X6b_DNA, and MER117, acquired distal enhancer activity for *Wnt5a* expression in the frontonasal region, including the palate shelves, during morphogenesis of the mammalian secondary palate [1]. As *Wnt5a* plays a key role in palatogenesis, it is likely that these TEs are involved in the complete closure of the secondary palate, which is a mammalian-specific trait. Second, he talked about his unpublished data showing that thousands of mammalian TEs contain binding sites for key transcription factors required for mammary gland development. Indeed, in mammary gland-derived cells, these regions contained chromatin signatures for enhancer activity. As this discovery is also related to a mammalian-specific trait, it underscores the role of TEs in mammalian morphological evolution via the modification of *cis*-regulatory networks.

Recent deep sequencing analyses have paved the way for a new research field of chromosome-associated long non-coding RNAs (lncRNAs), which play crucial roles in structural and epigenetic regulation during the mitotic cell cycle. However, little is known about the association of lncRNAs with meiotic chromatin in germ cells. **Ryusuke Nakajima** (RIKEN Institute, Japan) gave a talk on a mouse germline lncRNA (termed *R53*) that carries a SINE (short interspersed element) B1 motif. He showed that the *R53* RNA is expressed in adult testis and that it is a specific component of meiotic metaphase chromatin in mouse spermatogenesis. Knockdown of the *R53* transcript in mouse testis resulted in the misregulation of several postmeiotic genes and a significant reduction in the number of postmeiotic cells [2]. These findings indicate a role of the SINE-containing lncRNA in the regulation of chromatin structure and function during mouse spermatogenesis.

### TEs in gene innovation

While many examples of exaptation of TE-derived proteins have been accumulated to date, the following question remains largely unaddressed: What proportion of TE-derived proteins have acquired cellular functions? Furthermore, it is essential to elucidate the specific functions acquired by the exapted proteins as they exhibit several different functions. *Paternally expressed 11/retrotransposon-like 1* (*Peg11/Rtl1*) is an imprinted eutherian-specific TE-derived gene that is known to play an indispensable role in the maintenance of the placental fetal capillary network. **Moe Kitazawa** (Tokyo Medical and Dental University, Japan) explained her unpublished results that indicated that *Peg11/Rtl1* is also essential for muscle development. She showed that *Peg11/Rtl1* is expressed in fetal muscular cells and that the loss or the overexpression of *Peg11/Rtl1* causes different morphological abnormalities in fetal and neonatal skeletal muscles that lead to neonatal mortality. In humans, chromosome 14 carries the *PEG11/RTL1* gene. Uniparental disomy (UPD) of chromosome 14 causes muscle-related defects, suggesting that the misregulation of *PEG11/RTL1* in patients with maternal UPD(14) or paternal UPD(14) during embryogenesis is possibly responsible for the pathogenesis.

**Mahoko T. Ueda** (Tokai University, Japan) talked about a database of > 600,000 protein-coding endogenous viral elements (EVEs) for genomes of 19 mammalian species (available at gEVE database: http://geve.med.u-tokai.ac.jp) [3]. She showed that, while protein-coding EVEs (ppEVEs) are depleted near transcriptionally active sites, such as DNase I-hypersensitive and transcription start sites, there are > 1000 ppEVEs that encode proteins that are expressed in healthy human tissues (Ueda et al., *unpublished*). This indicates that further analyses of the expression and function of ppEVE-derived genes in multiple species is warranted to gain a better understanding of these unannotated genes and their role(s) in mammalian evolution.

Long interspersed nuclear element 1 (LINE-1) can also contribute to gene innovation via the generation of retrogenes, which are derived from the retrotransposition of various cellular mRNAs. **Kazuhiko Ohshima** (Nagahama Institute of Bio-Science and Technology, Japan) described his research on a young retrogene, *PIPSL*. This gene was created via a unique mechanism, whereby two tandemly arranged genes (a lipid kinase and a proteasome subunit) were assembled at the RNA level, and the resulting chimera was subsequently reverse transcribed and integrated into the genome of the common ancestor of extant hominid species using LINE-1 [4]. He showed that *PIPSL* is expressed in the testis of the white-handed gibbon and that the cluster of its transcription start sites in testes is conserved in gibbons, orangutans, and humans [5]. Although its precise function remains unelucidated, this unique gene has been conserved in structure and transcriptional regulation for approximately 20 million years.

### The mobilization of DNA transposons and its impact on genome evolution

Rice *mPing,* a miniature inverted-repeat TE (MITE), is an excellent model for studying rapid TE amplification (bursts) as it has attained high copy numbers (approximately 1000) in a short time period in the temperate *japonica* rice strain, Gimbozu EG4. **Takuji Tsukiyama** (Kindai University, Japan) talked about how *mPing* has attained such transposability. He found that most de novo *mPing* insertions arose in embryogenesis, usually 3–5 days after pollination (i.e., fertilization). During this period, the expression of *Ping*, a DNA transposon encoding a transposase that mobilizes the *mPing* sequence, was upregulated in EG4, whereas upregulation was not observed in an *mPing*-inactive cultivar, Nipponbare. Therefore, the rapid amplification of *mPing* in EG4 is possibly triggered by an early embryogenesis-specific derepression mechanism [6]. It would be interesting to elucidate the mechanism by which *Ping* escapes host-mediated transcriptional silencing during its upregulation.

DNA transposons excise their DNA sequences for mobilization; this process generates transposon footprints at the site of excision, which are often observed as insertions or deletions of a small number of nucleotides. When one cut-and-paste transposition is followed by another cut-and-paste transposition of the inserted transposon, termed as “paste-and-cut”, the site loses the transposon sequence. **Akihiko Koga** (Kyoto University, Japan) explained an interesting case of genetic modification caused by a DNA transposon. In an albino mutant of the medaka fish that carries the *Tol2* transposon in a pigment-related gene, spontaneous mutations with various phenotypes occurred at a high frequency [7]. Intriguingly, these arose through secondary mutations caused by the excision of the entire *Tol2* sequence accompanied by an insertion or deletion of a single or a small number of nucleotides at the excision site. It is noteworthy that unless the entire paste-and-cut process is monitored, no recognizable traces are left that indicate the participation of a transposon. These findings suggest that the small DNA insertions and deletions detected in related genes among different species could have been generated by an ancient “paste-and-cut” transposition. Even almost fossilized DNA transposons, such as the medaka *Tol1* element, are capable of undergoing transposition bursts [8]. Based on these findings, A. Koga proposed that DNA transposons, by acting as the major source of small DNA insertions and deletions, could have a more significant role in the evolution of their host genomes than was previously postulated.

### Regulation of TE mobility

The mobility of TEs is strictly regulated at transcriptional levels, RNA stability, and transposition. H3K9 methylation and Piwi-interacting RNAs (piRNAs) are often used for the repression of active transposons; however, their downstream effectors remain largely unknown. **Kei Fukuda** (RIKEN, Japan) presented recent results identifying cellular factors involved in H3K9me3-mediated silencing in mouse embryonic stem cells (mESCs). He used the genome-wide CRISPR screen system to identify candidate genes involved in the silencing of a GFP reporter in a Moloney murine leukemia virus (MLV)-based retroviral vector, which is repressed by the SETDB1*/*TRIM28 pathway in mESCs. Out of the > 80 genes identified, Fukuda and colleagues focused on MORC2A and RESF1 [9]. RESF1 (retroelement silencing factor 1) is a novel protein involved in retroelement silencing. He demonstrated that RESF1 anchors SETDB1 to the LTR to aid the local accumulation of H3K9me3, and therefore plays a role in maintaining the H3K9me3 modification. On the other hand, MORC2A appears to aid the spreading of H3K9me3. Furthermore, the identified proteins repress different types of retroelements: MORC2A targets young full-length LINE-1 copies, while RESF1 mainly represses endogenous retroviruses.

**Kuniaki Saito** (NIG, Japan) explained his unpublished study, demonstrating that CG14438 (a protein with zinc finger motifs) is required for transposon silencing in the *Drosophila* ovarian somatic cell line. Intriguingly, the increase in TE mRNA levels by CG14438 depletion was more than the increase caused by Piwi depletion, whereas CG14438 depletion did not affect piRNA abundance, suggesting that CG14438 is a TE silencing effector mediated by the Piwi-piRNA pathway. Notably, these findings are consistent with a recently published study by the Erdelyi laboratory [10].

**Taku Sasaki** (The University of Tokyo, Japan) discussed about a host–TE arms race in *Arabidopsis*, where *VANDAL21*, a DNA transposon, counteracted the host silencing system. In the wild-type plant, genomic *VANDAL21* copies are epigenetically silenced by DNA and H3K9 methylation; however, they are derepressed when one of its coding genes, VANC21, is introduced into the host as a transgene [11]. This anti-silencing effect depends on the VANC21 protein and is sequence-specific: only *VANDAL21* copies are activated while TEs from closely related families remain silenced. The VANC21 protein binds to DNA through a 9-bp sequence motif, and multiple binding sites are present in non-coding regions of the *VANDAL21* sequence [12]. These binding sites are often found within repetitive sequences, and are likely to have evolved in a concerted manner as the binding motifs have been specifically acquired by *VANDAL21*.

Another important question is whether and how the copy number of a particular TE affects the silencing pathway. **Hitoshi Nakayashiki** (Kobe University, Japan) discussed his unpublished studies on a relationship between the copy number and DNA methylation of the LTR retrotransposon *MAGGY* from the fungus *Pyricularia oryzae*. He found that *MAGGY* DNA was methylated in a copy number-dependent manner in the *P. oryzae* genome. *P. oryzae* RecA homologs, such as MoRad51 and MoRad55 were required for the copy number-dependent DNA methylation, suggesting that the homologous recombination machinery is involved in a link between copy-number counting and DNA methylation. **Yoko Ikeda** (Okayama University) reported new factors involved in TE silencing in plants, which were identified by the screening of *Arabidopsis* mutants that failed transgene silencing. One of these mutants is *mail1*, which encodes a protein with an aminotransferase-like plant mobile domain. She showed that specific TEs were upregulated in the *mail1* mutant, while the DNA methylation and siRNA levels of these upregulated TEs remained unchanged. The effects of *mail1* mutation were independent of the *ddm1*, *rdr2, mom1,* and *morc6*, suggesting that *mail1* is involved in a new silencing pathway [13]. Intriguingly, this protein is highly related to a group of TE-encoded proteins (*Gypsy*-type elements), suggesting that this TE silencing factor evolved through an association with TE.

### piRNA-related RNAs in diverse phenomena

Although piRNAs were possibly invented to silence TEs, they are involved in other cellular systems as well. The ciliated protozoan *Tetrahymena thermophila* contains approximately 12,000 internal eliminated sequences (IESs), many of which are related to TEs. IESs are eliminated from the somatic genome when the somatic macronucleus differentiates from the germline micronucleus. In this process, IESs are heterochromatinized through a piRNA-related mechanism and are eventually excised by a domesticated transposase. **Kensuke Kataoka** (National Institute for Basic Biology, Japan), whose research focuses on the mechanism of RNA-mediated heterochromatin formation and its higher-order assembly [14, 15], described his recent approaches to further understand the molecular links between heterochromatin components and DNA elimination. Kataoka and colleagues tethered all known heterochromatin proteins (HPs) and HP1-like proteins to an artificially created locus, which revealed that some non-HP1-like proteins, in addition to HP1-like proteins, are sufficient to induce DNA elimination at the tethered site (Kataoka et al.*, unpublished*). These findings suggest that RNA-guided DNA elimination is mechanistically divided into RNA-guided heterochromatinization and heterochromatin-guided DNA elimination.

Using a *Drosophila* model of amyotrophic lateral sclerosis (ALS), **Keiko Wakisaka** (Kyoto Institute of Technology, Japan) presented an interesting link between piRNA biogenesis and a neuronal disorder. Cabeza (*Caz*) encodes a nuclear protein responsible for ALS. Neuron-specific *Caz* knockdown in flies results in anatomical defects in motoneurons at neuromuscular junctions, leading to locomotion defects in flies that are similar to those observed in patients with ALS [16]. In addition, Wakisaka demonstrated that the Caz protein physically interacts with pre-piRNAs, and abnormal pre-piRNAs are abundantly produced in the central nervous system of the *Caz*-knockdown flies. Furthermore, overexpression of *Aub*, one of the Piwi genes, induced abnormal cytoplasmic accumulation of the Caz protein. These results support a model in which Caz binds to abnormal pre-piRNAs, and if these pre-piRNAs are in excess, Caz remains tethered to the cytoplasm in an *Aub*-dependent manner [16].

## Concluding remarks

Recent studies have shed new light on the roles of TEs in various host biological processes, including neurogenic function, placental function, pluripotency, transcriptomic regulation, and nuclear spatial organization. With new sequencing technologies, TEs are no longer the “dark matter” of the genome, and we anticipate that in the near future the regulation mechanism of individual TE copies and their involvement in the regulation of other parts of the genome will be addressed. The next meeting would be held in 2020, and we hope that this series of meetings would continue to enhance TE studies in Japan and other Asian countries.

## Abbreviations

ALS: Amyotrophic lateral sclerosis
EVE: Endogenous viral elements
HP: Heterochromatin protein
IES: Internal eliminated sequence
LINE: Long interspersed element
lncRNA: Long non-coding RNA
mESC: Mouse embryonic stem cell
MITE: Miniature inverted-repeat transposable element
MLV: Moloney murine leukemia virus
piRNA: Piwi-interacting RNA
SINE: Short interspersed element
TE: Transposable element
UPD: Uniparental disomy
